# Abstract of the Proceedings of the Buffalo Medical Association

**Published:** 1862-12

**Authors:** J. F. Miner

**Affiliations:** Secretary


### BUFFALO

### Medical and Surgical Journal

VOL. II. DECEMBER, 1862. NO. 5.

### ORIGINAL COMMUNICATIONS.

ART. I.—*Abstract of the Proceedings of the Buffalo Medical Association.*

TUESDAY EVENING, November 4th, 1862.

Prof. James P. White, President, in the Chair.

*Dr. Miner* presented a uterine polypus which he had the day before removed from the uterus of a patient twenty-eight years old, married for four years, but absent from her husband until the last two years. Had never had children, but for the last three months the menstrual secretion had not appeared, and she mistrusted that she was now pregnant. A few days since she presented herself for advice on account of slight hemorrhage from the vagina, and somewhat profuse leucorrhœal discharge. Upon examination, the tumor was distinctly in view, occupying the cervical canal, which was greatly dilated, and the neck of the uterus enlarged and flaccid. It was the size of a large grape, somewhat elongated and flattened, and attached to the posterior surface of the cervical canal, near the upper portion, its body and neck reaching to the vagina. The tumor was removed by torsion, and the surface penciled by the crayon of nitrate of silver, to arrest hemorrhage and guard against reproduction.—The tumor consisted largely of cellular tissue, was covered by mucous membrane, and supplied with delicate vessels. It was surrounded by, or imbedded in, a large amount of tenacious transparent albuminous matter, which might be still seen, adhering to the pedical in considerable quantity. This is a representative specimen of this form of polypus, which is by no means of infrequent appearance, and described by authors as mucous polypus.

The only question of especial interest in connection with the removal, is, should such operation be made while there is reason to suppose the patient pregnant? Or, should it be delayed until after parturition from fear of the disturbance producing abortion? His own opinions were sufficiently manifest by the action he had taken. Others, holding to different views, were at liberty to answer the inquiry.

Dr. M. also related the case of a young married woman, mother of three children, who had always enjoyed most perfect health until quite recently. She commenced to have some leucorrhœal discharge, and soon blood mixed with the mucus, and at last profuse hemorrhage from the vagina, which did not much intermit, but had continued constant with occasional exacerbations, when a pint of blood would be discharged in a few minutes. This greatly reduced the strength, and when applying for advice the patient was sufficiently alarmed to allow vaginal examination without objection. The cervical canal was enlarged and patulous, and a great number of cysts were plainly visible, both within the canal and also around the os-uteri. These vesicles varied in size from one line in diameter to three or four, and could be easily broken down and made to disappear by pressure with an instrument having a roughened surface; some were broken down by the uterine forceps used to dilate the os-uteri for the purpose of better observation.—The uterus was found to be considerably enlarged, the uterine sound passing readily some four and a half or five inches, and turning freely in all directions, showing great dilation of the uterine cavity. The only symptoms which induced the patient to seek for medical aid were the leucorrhœa and hemorrhage; other general indications of uterine disease were mostly wanting. Solid nitrate of silver was applied to the surface upon which the vesicles rested, every five days for three or four applications, when all hemorrhage ceased, the leucorrhœal discharge yet continued, the same application was made at longer intervals until all discharge abated. The cervical canal contracted to its natural diameter; the uterus also greatly diminished in the length of its internal cavity, and the patient was dismissed after five or six weeks' treatment, apparently well. The perfect success of this treatment is perhaps worthy of note, since it is generally regarded as inefficient in the removal of the disease. To increase its efficacy scarification has been suggested and adopted with the view of emptying the vesicles of their albuminous contents, and allowing the better application of the nitrate of silver upon the secreting surface; this plan, it is claimed, has been attended by marked success.

Deep scarifications were objected to as unnecessary, since the vesicles are generally very easily broken down, and when broken and the contents discharged, the abundant application of the solid nitrate of silver to the secreting membrane appeared to be quite efficacious.

Having spoken of these two forms of what are called mucous polypi he would also in this connection speak of a case which came under his care a few months since allied to these in the symptoms to which it gave rise, yet differing quite widely in its essential nature. A married lady, thirty-five years of age, the mother of four children, had ceased to menstruate for three months; the lower abdomen commenced to enlarge, breasts to swell, and she regarded herself pregnant. At about the fourth month she was slightly "unwell," that is, had slight discharge of blood from the vagina and after this she had similar attacks at irregular intervals, the abdomen continuing to increase, and the motions of the child thought to be felt.—Bearing down pains were now constant and hemorrhage very frequently profuse; the natural period of labor approaching, as she supposed. Her family physician was consulted, and opium and gum kino prescribed with the encouragement that he should soon be called to attend her in labor.—The expected period came and passed; pains were annoying, but not severe enough to require attendance of physician or to certainly indicate labor; contractions of the uterus were distinctly felt. Family physician was again consulted and prescribed something as before, evidently supposing that it was only a little miscalculation as to the period of gestation. Patient became nervous and anxious, was greatly reduced in blood and strength, and obliged to remain in bed, suffering with constant hemorrhage and considerable pain; motions of child or feelings as of the motions of child abated. At this period, ten months after first indications of derangement of the health Dr. M. was invited to visit the patient. Examination per vagina by the touch, detected tumor of the uterus partly projecting into vagina, attached by a pedicle to the uterus, or high up in the cervical canal. It was an inch and a half in its short, and two inches in its long diameter, rounded, rough and hard. A large speculum was introduced, and with long uterine forceps the neck was grasped, and after giving it a few turns separated and removed. The hemorrhage was considerable, but not dangerously profuse, and for five or six days tenderness and pain were present in the lower abdomen; after this the patient made rapid recevery. Upon examining the tumor it was found to be made up of distinct layers, differing in density and color, the outer being stronger and lighter color, while the center was deep red and granular. These outgrowths or productions of the uterus he mistrusted were quite rare, and at the time he removed it, he was unable to decide its character. So far as he has been able to learn, the first complete description of this affection was made by the late Prof. Kiwisch, in 1846. And they are now recognized and described under the name of fibrinous polypi, though this epithet is regarded as inappropriate. In certain conditions, independent, as Prof. Kiwisch believes, of impregnation; consequent, as others think, upon previous abortion, the walls of the uterus may be so soft and yielding as to allow of the gradual accumulation of effused blood in the cavity of the organ. In course of time the clot may not only pass through those changes by which the coloring matter is removed from its exterior, which assumes a dirty white or greyish aspect, while portions of a dark red hue are to be found within, but may also be the seat of the same kind of imperfect organization as has been observed in cases of hemorrhages into the arachnoid or of blood effused in other situations. Like cardiac polypi, so these become firmly adherent to the walls of the cavity, within which they form." These growths resemble in structure the contents of an old aneurismal sac; and the one removed possessed this appearance in very marked degree.

There are many questions connected with the conditions under which such hemorrhage occurs, and differences of opinion as to the causes which may produce it, but the object of all treatment would be the same, namely, to empty the uterus and remove the product, and afterwards to maintain the contracted state of the organ.

These cases had been stated in connection and at considerable length, and at once opened up many points of inquiry which are worthy of investigation. The histories of these cases are also of some interest, suggestive of the importance of thorough investigation previous to entering upon the treatment of uterine affections, and should put us upon our guard so that we do not lose our reputations and at the same time our patients by neglecting the common available means of correct diagnosis, and contenting ourselves by the most superficial examination.

There can be no special difficulty in the diagnosis of these larger out growths, since we have only to look, when their presence and character can be sufficiently known. The vesicular, or cystic growths, require a more careful observation, and when new and small might escape detection, especially if only present in the cervical canal. Tumors while retained within the uterine cavity are much more difficult to detect, and in some cases, previous to expulsion their presence cannot be positively ascertained, but will be very strongly inferred from the general symptoms; with persevering effort, such growths may almost always be diagnosticated while occupying the uterine cavity.

*Prof. White* remarked that he was very much obliged to the Secretary, Dr. Miner, for introducing the subject of uterine polypi, and also for presenting the pathological specimen he had exhibited. He had covered a wide field, and suggested many questions of interest. Yet, it would be impossible to speak of all, or indeed, of but very few of the many topics which the broad subject of uterine polypi at once suggested. To speak briefly of ordinary mucous polypi he would say that they were quite common, and that he had often found them as had been described, imbedded in mucous or albuminous fluid. As to the propriety of removal during pregnancy, he would say that the only danger of their remaining is from hemorrhage, and this is considerable, and is greatly increased by the pregnancy. Ordinarily, the danger is less to remove them, and it is generally proper, while if there is hemmorrage, it is imperative; they must be removed. The removal is not a formidable operation ; had done so in pregnancy without disturbing the process. The manner of removal he regarded of not much importance since any of the various methods would be successful. The pedicle being divided in these growths, it is fully demonstrated that the removal is complete ; the remaining portion of pedical is absorbed and the operation successful.

The other form of disease, the vesicular or cystic growths, he had also often observed. He had applied nitrate of silver and sponge dipped in solution of persulphate of iron. The best plan is to remove with the curved curett and afterwards apply the nitrate of silver. They usually accompany endo-metritis, and the secrētion is serous, sero-sanguinolent, or sanguinolent, and sometimes very profuse.

He was sent for to see a woman in Canada, who thought she would die from the hemorrhage. She had been under the care of several physicians without relief. The bleeding was so profuse he thought best to plug the vagina, and a kite-tailed tampon was introduced through the speculum. The tampon was not removed, and the patient came to Buffalo for his constant attendance. When on the second day the tampon was removed the hemorrhage had ceased in great degree, and great numbers of these vesicular growths were removed, scraped off with the curved curett, the surface penciled with persulphate of iron. The patient was fully restored after losing great quantities of blood.

*Dr. White* also related the following case: Mrs. B., mother of four children, thought that one year since she had abortion. She had violent pains, very much like first stage of labor, which had continued for some days, and profuse mucous leucorrhœa. Her attendant, a Homœpathist, informed her that there was "something the matter." Came next day, but made no investigations and told her nothing more definite. Oct. 19th, pains continuing, Dr. W. was invited to visit the patient. Found that from the uterus had been expelled a firm fibrous tumor. Assisted by Drs. Eastman, Mason and Whitmore, the ecraseur was applied and tumor removed. The next day she was examined; the neck of uterus was not very large, and another tumor was not suspected. The pains did not abate, and two days after removal of the first, a second tumor, larger than the first, was found and removed in like manner. It was as large as the egg plant. After this, had no farther pain or discharge; the uterus contracted, and on the 23d and 24th she felt pretty well; on the 25th she complained that her face felt numb, and had pain in her lip. Evening—she could not open her mouth; pulse not disturbed. Anodynes were given. She had spasms which he yet hoped were hysterical. 26th—head drawn back, mouth closely shut—in a word—had tetanus which he had never before seen follow any operation upon the uterus; died that evening in tetanic spasm.

This condition might have been induced by the protracted labor, and the irritation of the system, caused by so much pain.

*Dr. Cronyn* spoke of a case of menorrhagia; patient had passed two months without menstruation, and thought herself pregnant, when hemorrhage commenced. Prescribed persulphate of iron and ergot. In two weeks she was taken again with profuse bleeding, and after two weeks more she regained her strength so as to go to New York, when she was taken ill again and visited by Dr. Flint, who prescribed persulphate of iron also; she came home and he again made the same perscription. Digital examination satisfied him of the patulous and roughened condition of the neck of the uterus. He now refused all further care of the case without vaginal examination, which was allowed, and showed a roughened and abraded surface; the bleeding surface was distinctly seen; made free applieation of nitrate of silver and she soon after recovered.

*Dr. Samo* desired to suggest the propriety of physicians advancing upon usual charges in view of the depressed value of the paper dollar—one dollar per visit not being, in reality, more than sixty-two cents. Only wished to suggest this for the consideration of the society.

A short discussion upon this subject was participated in by the physicians present, and its further consideration postponed for future action.

Adjourned to the first Tuesday evening in December.

J. F. MINER, Secretary.

NOTE.—Mr. Editor:—In your report of the proceedings of the Buffalo Medical Association for October, you say that "Dr. Cronyn spoke of the seminal fluid upon the side of the egg being often streaked with blood," &c.; it should have been that he was struck with the fact of frequently observing a spot of blood upon said fluid, and whether or no there might not occasionally be a like spot upon human spermatozoa, and be developed *pari passu* with the fœtus in utero, and thus nevi &c. be readily accounted for. Again, when speaking of pelvic abscesses you say, "Dr. Cronyn said that Mr. Hilton in his lectures upon conservative surgery," &c., when it should be that the said Mr. Hilton in his lectures before the College of Surgery, England, when speaking of abscesses, advocated the making of a small incision through the skin with the lancet in the most dependent part, and then with a pair of dressing forceps passed into the wound tear open the sac of the abscess, and thereby save the accidents that frequently happen where a cutting instrument is used. By making the above corrections you will oblige

Yours, &c. JNO. CRONYN, M. D.

